# Building on the Enriched Hierarchical Model of Achievement Motivation: Autonomous and Controlling Reasons Underlying Mastery Goals

**DOI:** 10.5334/pb.281

**Published:** 2016-07-13

**Authors:** Aikaterini Michou, Lennia Matos, Rafael Gargurevich, Burcin Gumus, Dora Herrera

**Affiliations:** 1 Graduate School of Education Bilkent University 06800 Ankara, TR; 2 Pontifical Catholic University of Peru, PE; 3 Bilkent University, TR

**Keywords:** need for achievement, fear of failure, needs satisfaction, achievement goals, autonomous reasons, controlling reasons

## Abstract

Two motivational theories – the Achievement Goal Theory and Self-Determination Theory – have recently been combined to explain students’ motivation, making it possible to study the “what” and the “why” of learners’ achievement strivings. The present study built on this approach by (a) investigating whether the distinction between autonomous or volitional and controlling or pressuring reasons can be meaningfully applied to the adoption of mastery-avoidance goals, (b) investigating the concurrent and prospectiverelations between mastery-avoidance goals and their underlying reasons and learning strategies when mastery-approach goals and their underlying reasons were also considered, and by (c) incorporating psychological need experiences as an explanatory variable in the relation between achievement motives (i.e., the motive to succeed and motive to avoid failure) and both mastery goals and their underlying reasons. In two Turkish university students samples (*N* = 226, *M_age_* = 22.36; *N* = 331, *M_age_* = 19.5), autonomous and controlling reasons appeared applicable to mastery-avoidance goals and regression and path analysis further showed that mastery-avoidance goals and their underlying autonomous reasons fail to predicted learning strategies over and above the pursuit of mastery-approach goals and their underlying reasons. Finally, need experiences were established as mediators between achievement motives and both mastery goals and their underlying reasons.

Motivation is an important predictor of students’ quality of learning. Research carried out by Willy Lens and his associates has indicated that an enriched understanding of students’ motivation in a specific learning situation can be achieved if both students’ achievement goals and their underlying reasons for pursuing these achievement goals are considered (Vansteenkiste, Lens, Elliot, Soenens, & Mouratidis, 2014). Moreover, to fully account for students’ achievement motivation, personal and contextual motivational factors need to be taken into consideration (Elliot, 1999; Michou, Mouratidis, Lens, & Vansteenkiste, 2013).

In two university student samples, we investigated to what extent students’ goal to learn as much as possible (i.e., mastery-approach goal) and their goal to avoid learning less than it is possible (i.e., mastery-avoidance goal) can be endorsed for pressuring or controlling and for volitional or autonomous reasons. Previous research has shown that mastery-approach goals can be pursued for either autonomous (e.g., interest, enjoyment) or controlling (e.g., self-worth concerns, social approval) reasons, resulting in positive and negative outcomes, respectively (Benita, Roth, & Deci, 2013; Gaudreau, 2012). Would this distinction between autonomous and controlling reasons also apply to mastery-avoidance goals, with similar implications for learners’ outcomes? This question, which has not been investigated yet, will be addressed in the present study.

To obtain a full understanding of students’ achievement motivation, we also examined its contextual and personal antecedents. Specifically, we investigated whether students’ achievement motivation (i.e., mastery-approach and mastery-avoidance goals and their autonomous and controlling underlying reasons) is rooted into their (a) experiences of need satisfaction and need frustration, and (b) their personal tendency to approach success (i.e., motive to succeed) or to avoid failure (i.e., motive to avoid failure).

## The Achievement Goal Approach: “What” of Achievement Striving

Achievement goals have been initially been broadly defined as they involved both a specific aim and a reason (i.e., the purpose) for individuals’ achievement strivings (Maehr, 1989). Within this broader conceptualization, two different achievement goals have been identified; the goal to develop competence either through self-improvement or through mastering the requirements of a task (referred to as a mastery, learning or task goal) and the goal to demonstrate one’s ability through outperforming others (referred to as a performance or ego-goal; Ames, 1992; Dweck & Leggett, 1988; Nicholls, 1984).

Depending on whether competence was positively or negatively valenced, these two achievement goals were subsequently differentiated into those characterized by an approach orientation toward success and those with an avoidance focus to obviate failure (Elliot & McGregor, 2001). Specifically, mastery goals, upon which we focused in the present study, got divided into *mastery-approach goals* (MAp) – those that orient individuals to meet some task-based or self-based criteria to achieve success – and *mastery-avoidance goals* (MAv) – those that orient individuals towards the avoidance of task-defined or self-defined failure (Elliot & McGregor, 2001).^1^

The idea to consider the approach and avoidance aspect of the achievement goals was congruent with the formulation of a hierarchical model of achievement motivation according to which several personal or contextual factors influence the endorsement of specific achievement goals and, through them, the patterns of learning (Elliot, 1999). The most studied antecedents of achievement goals have been the dispositional achievement motives to succeed and to avoid failure. The motive to succeed, an appetitive tendency acquired in early age, has been linked with MAp, whereas the motive to avoid failure, an inhibitory tendency acquired also during early childhood, has been linked with MAv goals (Elliot, 2005).

Research has shown that MAp goals, once instigated by the motive to succeed, then direct behavior to attain mastery and learning; hence, MAp goals are correlated with positive educational outcomes, such as students’ intrinsic motivation and effective learning strategies (Bartels & Magun-Jackson, 2009; Elliot & McGregor, 2001). Following this logic, one could expect the MAv goals, which are characterized by a negative (i.e., the avoidance tendency) and a positive (i.e., mastery attainment and personal improvement) feature, to predict a mix of positive and negative outcomes. Yet, most previous studies found MAv goals to be related to negative outcomes only. For example, MAv goals are negatively related to performance improvement (Van Yperen, Elliot, & Anseel, 2009) and positively related to procrastination, surface processing, and disorganization (Howell & Watson, 2007) and anxiety (Elliot & McGregor, 2001). Only very recently, Senko and Freund (2015) found that MAv goals among older adults can be perceived as attainable and related positively with task enjoyment. It seems that the (mal)adaptive nature of this type of achievement goal deserves further investigation. We did so in the present study, thereby comparing them to the more adaptive MAp goals.

Another reason why the study of MAv goals also deserves greater attention is due to some recent developments in the achievement goal literature. Because Elliot (2005) suggested restricting the definition of achievement goals to aims only (instead of involving both aims and reasons), researchers have begun to systematically study different combinations of the “what” of one’s achievement striving (i.e., the goal aims) with a variety of motivational forces that are related to the “why” of goal striving (i.e., the goal reasons) (Dompnier, Darnon, & Butera, 2009; Urdan & Mestas, 2006). In this context, Lens, Vansteenkiste and collaborators conceived the reasons underlying achievement goals from the perspective of Self-Determination Theory (Vansteenkiste, Lens et al., 2014). We believe that this differentiated approach is very suitable to further examine the (mal)adaptive nature of the less studied MAv goals. Would the correlates of this “hybrid of positive (mastery) and negative (avoidance) motivational forces” (Senko & Freund, 2015, p. 477) depend on the autonomous or controlling reasons for endorsing this achievement goal?

## Self-Determination Theory: “Why” of Activity Engagement

According to Self-Determination Theory (SDT; Ryan & Deci, 2000), people regulate their behavior using a variety of reasons that could be categorized as either controlling or autonomous in nature. The controlling category includes those reasons that involve pressure, which can either come from oneself (e.g., feelings of guilt, shame) or from the external environment (e.g., threats, rewards). The autonomous category includes those reasons that are in accordance with the self, including a personal interest in and enjoyment of an activity or the personal endorsement of its importance.

In the educational domain, research has shown that autonomous motivation is related to various adaptive outcomes such as deep learning strategies and effort (Kusurkar, Ten Cate, Vos, Westers, & Croiset, 2012), academic and social competence, academic performance (Soenens & Vansteenkiste, 2005), prosocial behavior (Ryan & Connell, 1989), and adjustment (Black & Deci, 2000). In contrast, controlled motivation is related to maladaptive educational outcomes such as maladaptive coping strategies, low academic performance, superficial cognitive processing and dropout.

According to the SDT (Deci & Ryan, 2000), the prerequisite for autonomous motivation to unfold is the satisfaction of three innate psychological needs. When people satisfy their needs for autonomy (i.e. the need to experience oneself as the agent of one’s experience), competence (i.e. the need to feel effective in the interaction with the environment) and relatedness (i.e. the need to feel connected with and related to others), they are more likely to behave on the basis of autonomous motives. In contrast, when people frustrate their needs for autonomy, competence, or relatedness get frustrated, they feel coerced to participate in an activity, incompetent to deal effectively with it, and disconnected from others. In such cases people are more likely to participate in an activity driven by controlling motives or they display amotivation, that is, they have little if any intention to execute the activity at hand (e.g., Haerens, Aelterman, Vansteenkiste, Soenens, & Van Petegem, 2015).

## “What” and “Why” of Achievement Goals

Although the autonomous-controlled motivation distinction has been primarily used to consider the reasons underlying individuals’ activity engagement (e.g., Ryan & Connell, 1989), this distinction can be equally applied to individuals’ goal pursuit in general (e.g., Sheldon & Kasser, 1998) and their achievement goal pursuit in particular (e.g. Vansteenkiste, Smeets et al., 2010). To illustrate the latter, learners can be mastery-approach oriented because they find it challenging, interesting or personally important to fully master the requirements of a task (autonomous motivation) or because they would feel guilty or less worthy if they did not put effort and succeed in mastering the task (controlled motivation; Benita et al., 2014). Lens, Vansteenkiste and collaborators (Vansteenkiste, Mouratidis, & Lens, 2010) developed this argument and proposed that autonomous and controlling reasons (the “why”) can undergird the endorsement of achievement goals (the “what”).

This line of research, conducted in both the sport (Delrue et al., this issue; Gaudreau & Braaten, this issue; Vansteenkiste, Mouratidis, Van Riet, & Lens, 2014) and educational domain (Benita, et al., 2014; Gaudreau, 2012; Michou, Vansteenkiste, Mouratidis, & Lens 2014) suggested that the autonomous reasons underlying MAp goals were positively associated with academic satisfaction (Gaudreau, 2012), performance, challenge appraisals (Delrue et al., this issue), intrinsic motivation (Benita et al., 2014) and prosocial behavior (Vansteenkiste, Mouratidis et al., 2014). On the other hand, controlling reasons underlying MAp goals were negatively related to effort regulations (Michou, Vansteenkiste et al., 2014).

In most of these studies, MAp goals significantly predicted the outcomes when the autonomous or controlling reasons were also considered (e.g., Gaudreau, 2012; Michou, Vaanstenkiste et al., 2014). This suggests that both achievement goals and underlying reasons can predict a unique portion of the variation in student outcomes. In addition, in some of these studies interactions between MAp goals and underlying reasons were found (e.g., Gaudreau, 2012; Gaudreau & Braaten, this issue) such that strong endorsement of MAp goals, coupled with autonomous reasons, related to even more positive outcomes. However, in none of these studies the relation of MAv goals and their underlying reasons to outcomes were examined and none of them made use of a longitudinal design to examine the relation of mastery goals and underlying reasons to outcomes when controlling for outcomes at baseline level. We addressed both these issues in the present study.

## The Antecedents of the “what” and “why” of Achievement Goals

As concerns the antecedents of individuals’ achievement goals and underlying reasons, Michou, Vansteenkiste et al. (2014) considered students’ motive to succeed and their motive to avoid failure as their dispositional antecedents (see also Michou, Matsagouras, & Lens, 2014). They found that the motive to succeed was positively related to approach-oriented achievement goals and autonomous reasons, whereas the motive to avoid failure was positively related to avoidance-oriented achievement goals and controlling reasons. By incorporating the reasons underlying achievement goals (apart from the achievement goals as such), they enriched the hierarchical model of achievement motivation. We aimed to build on this line of research by examining whether need-based experiences, that is, both the satisfaction and frustration of the psychological needs for autonomy, competence, and relatedness, would serve to explain the relation between the dispositional achievement motives and the achievement goals and their underlying reasons.

## The Present Study

For the purpose of the present study, two samples of Turkish learners were recruited. Sample 1 was cross-sectional in nature, whereas Sample 2 involved a short-term prospective design, with students’ learning strategies being assessed at the beginning and at the end of a trimester. The present study had three aims. First, we investigated whether autonomous and controlling reasons are applicable to the endorsement of MAv goals (i.e., the aim to avoid learning less than it is possible). Although MAv goals may be rarely endorsed by young adults for autonomous reasons, some may find it challenging or even enjoyable to avoid learning less than it is possible. Specifically, if the goal is to avoid reaching less than one’s potential, such a goal can be challenging and interesting (i.e., autonomously motivated) but also can be undergirded by the avoidance of guilt and criticism (i.e., controlled motivated) for both young and mature adults. For this reason, we hypothesized that MAv goals can be endorsed for either autonomous or controlling reasons as reflected by a different percentage of students that will score high in autonomous and controlling reasons underlying MAv goals.

Second, we investigated the relation of MAv goals and their underlying reasons with learning strategies, while controlling for MAp goals and their underlying reasons. We hypothesized that MAv goals may initially relate to learning strategies, but once their underlying reasons and MAp goals are considered, only MAp goals and MAv autonomous reasons will positively relate to learning strategies. We made this hypothesis based on Michou, Vansteenkiste et al., (2014), who found only MAp goals and autonomous underlying reasons to predict effective learning strategies when controlling for PAp and PAv goals. Moreover, in an integrated model with the autonomous and controlling reasons underlying the MAp goals included, we examined whether the reasons underlying MAv goals would remain significant predictors. In this integrated model, we hypothesized that MAp goals and its underlying autonomous reasons would yield the greatest power in predicting learning strategies. We tested this hypothesis both at a cross-sectional level (i.e., Sample 1) and prospectively (i.e., six weeks later; T2) thereby controlling for learning strategies at Time 1 (i.e., Sample 2). This allows us to overcome the potentially inflated correlations that may exist between learning strategies and the MAp/MAv goals and their underlying reasons at a single point of time (see also Gillet, Lafreniere, Vallerand, Huart, & Fouquereau, 2012).

Our third aim was to investigate the relation of MAp and MAv goals and their underlying reasons with dispositional achievement motives and experiences of need satisfaction and need frustration. Based on Sheldon’s (2011) two-process model that links dispositional motives and psychological needs, we considered individuals’ psychological need-based experiences as intervening variables in between the more general motive to succeed or the motive to avoid failure and the “what” and “why” of achievement goals. Consistent with this argument Schüler, Sheldon, and Fröhlich (2010) found competence need satisfaction to mediate the relation between need for achievement and situational motivation to attain a goal. Building on this research, herein we deemed it important to consider the satisfaction or frustration of all three needs (i.e., autonomy, competence, and relatedness) as all of them are considered to contribute to personal growth (Deci & Ryan, 2000).

**Figure 1:**
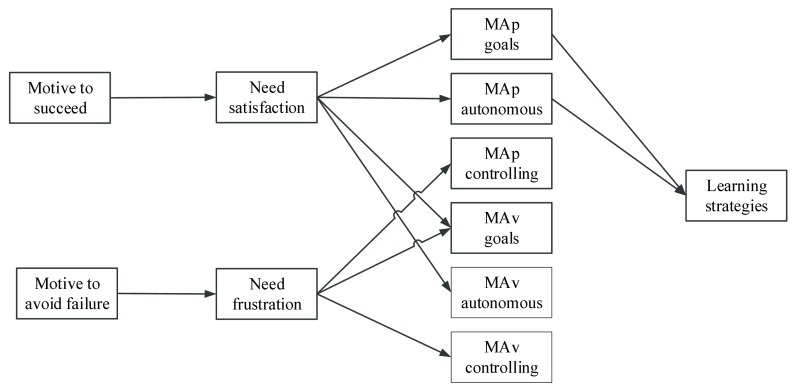
The Hypothesized Model.

Specifically, we hypothesized that the motive to succeed will relate positively to need satisfaction, whereas the motive to avoid failure will relate positively to need frustration. This is because the motive to succeed is a disposition developed through enduring experiences of success, which can be also considered as the necessary condition for an individual to develop an agentic propensity in schooling [i.e. a tendency to seek for or to create the necessary conditions for need satisfaction (Reeve, 2013)]. Regarding the motive to avoid failure, as this avoidance disposition reduces students’ engagement in an attempt to avoid failure, it can also reduce students’ feelings of competence, autonomy and relatedness. Further, we hypothesized that need satisfaction and need frustration would be, respectively, related to autonomous and controlling reasons underlying both MAp and MAv goals. Finally, we hypothesized that need-based experiences would also relate to the adoption of specific achievement goals. Specifically, research has shown that need satisfaction is positively related to MAp goals (Diseth, Danielsen, & Samdal, 2012; Janke, Nitsche, & Dickhauser, 2015) and that autonomy and relatedness satisfaction are related to both MAp and MAv goals (but not to PAp or PAv goals) (Ciani, Sheldon, Hilpert, & Easter, 2011). As Deci and Ryan (2000) pointed out, mastery goals are more aligned with intrinsic motivation when compared to performance goals. Therefore, mastery goals are more probably related to need satisfaction. Thus, we assumed that need satisfaction will relate to both MAp and MAv goals, but, given the less adaptive nature of MAv goals, we did not exclude the case that MAv goals could also be related to need frustration. An overview of the hypothesized model can be found in Figure 1.

## Method

### Participants and Procedure

Sample 1 (i.e., the sample of the cross-sectional study) included 226 (*M*_age_ = 22.36, *SD* = 3.92; 67.4% females) students from a private non-profit university in an urban area in Turkey. Sample 2 (i.e., the sample of the short-term prospective study) consisted of 331 students (N = 284 for T1 and N = 196 for T2 among whom 38 were not included in T1 and 9 did not report their learning strategies; *M*_age_ = 19.50, *SD* = 1.50; 54% females; 27 students omitted reporting their age and 33 did not report their gender) from an English language preparatory program of the same university. As for Sample 2, 158 students participated in both T1 and T2 assessments (42.50% attrition), missing value analysis showed statistically non-significant pattern (Little MCAR test, x^2^(62) = 65.69, *p* = .35). Both studies were approved by the ethical committee of the University. The questionnaires were applied during a class session. Before entering each class, research assistants asked class instructors for permission. Students consented to participate after being informed that they could quit the session at any point if they would like to do so. Students in Sample 2 also reported their learning strategies in the same class at the end of a trimester (i.e., six weeks later). The various measures were identical for both samples, and independently translated from English to Turkish by two experts in the field, with discrepancies being resolved according to the procedures proposed by Hambleton (1994). The participants completed the survey by using five point-Likert type scales.

### Measures

**Motive dispositions.** We used the short-version of the Achievement Motivation Scale (AMS; Lang & Fries, 2006) to assess the motive to succeed (5 items; e.g., “I like situations in which I can find out how capable I am”; α = .87 and .92 for Sample 1 and Sample 2, respectively) and the motive to avoid failure (5 items; e.g., “Even if nobody would notice my failure, I’m afraid of tasks which I’m not able to solve”; α = .86 for Sample 1 and α = .85 for Sample 2).

**Need satisfaction and need frustration.** The balanced measure of psychological needs questionnaire (BMPN; Sheldon & Hilpert, 2012) was adapted to assess students’ need satisfaction and frustration regarding their studies. Satisfaction of each psychological need (i.e. autonomy, competence and relatedness) was assessed by three items (e.g., “I was free to do things my own way” *for autonomy*; ‘I took on and mastered hard challenges” *for competence*; “I felt close and connected with other people” *for relatedness*), and so was frustration of each need (e.g., “I had a lot of pressures I could do without” *for autonomy*; “I struggled doing something I should be good at” *for competence*; “I felt unappreciated by one or more important people” *for relatedness*). To create a need satisfaction composite score, the nine items for autonomy, competence, and relatedness need satisfaction were averaged (α =.77 for Sample 1 and α = .74 for Sample 2) and the same was done for the nine items for autonomy, competence and relatedness need frustration in order to create a need frustration composite score (α =.78 for Sample 1 and α = .79 for Sample 2).

**Achievement goals.** To assess students’ mastery goals, in both samples two items of the Revised Achievement Goal Questionnaire (AGQ –R; Elliot & Murayama, 2008) were used. These two items represented a mastery-approach goal (e.g., “My goal in this course is to learn as much as possible”) and a mastery-avoidance goal (e.g., “My goal in this course is to avoid learning less than it is possible to learn”).

**Underlying reasons of achievement goals.** This study followed the operationalization of Vansteenkiste, Mouratidis and collaborators (2010) to assess students’ autonomous and controlling reasons underlying the pursuit of their mastery goals. After the presentation of each mastery goal item, eight reasons were listed for pursuing the goals. Participants assessed the reasons for pursuing the mastery goals only in the case that they had scored equal or higher than three in the mastery goal item. Of these eight items, (a) two assessed intrinsic reasons (e.g., “I found it a challenging goal to pursue”), (b) two assessed identified reasons (e.g., “I found it a personally important goal”), (c) three items assessed introjected reasons (e.g., “I needed to prove it to myself”), and (d) one item assessed external reasons (e.g., “Others (teacher, parents) obliged me to do so”).

In the assessment of the underlying reasons three methodological advantages were applied compared to previous research: (a) we asked participants to report the underlying reasons only if they highly endorsed the MAp or MAv goal, (b) we used only one item for each goal so that the reasons for each goal would be presented only once, and (c) we changed the order of presentation to the reasons items after each goal. This allowed us to minimize the potential for inflated correlations between the MAp autonomous and MAv autonomous, as well as between the MAp controlling and MAv controlling reasons (as it appeared in Michou, Vansteenkiste et al., 2014).

Principal component analysis for the reasons underlying MAp and MAv goals across samples extracted two factors when the external reason was excluded. The first factor represented the controlling reasons including the three introjected items (Lambda varied between 3.85 and 1.14 with the explained variance varying between 37.59% and 26.01%), and the second factor represented the autonomous reasons including the two intrinsic and the two identified reasons (Lambda varied between 3.11 and 0.82 with the explained variance varying between 34.63% and 27.92%). Based on this result, we computed a composite score for controlling reasons underlying both MAp (α = .79 for Sample 1 and α = .67 for Sample 2) and MAv goals (α = .79 for Sample 1 and .74 for Sample 2) by aggregating only the three introjected reasons. The two intrinsic and the two identified scores were also aggregated to create a composite score for autonomous reasons underlying both MAp (α =. 69 for Sample 1 and α = .75 for Sample 2) and MAv pursuit (α = .76 for Sample 1 and α = .80 for Sample 2).

**Learning strategies.** Parts of the Motivated Strategies for Learning Questionnaire (MSLQ, Pintrich, Smith, Garcia, & McKeachie, 1993) was administered to assess three aspects of students’ learning strategies. Specifically, students reported their use of (a) critical thinking (5 items; e.g., “I often find myself questioning things I hear or read in this course to decide if I find them convincing”; α= .74 for Sample 1 and α = .73 in T1 and α = .72 in T2 for Sample 2), (b) meta-cognitive self-regulation (5 items; e.g., “When I become confused about something I’m reading for my class, I go back and try to figure it out”; α = .75 for Sample 1 and α = .72 in T1 and α = .81 in T2 for Sample 2); and (c) effort regulation (4 items; e.g., “I work hard to do well in this class even if I don’t like what we are doing”; α = .64 for Sample 1 and α = .67 in T1 and α = .62 in T2 for Sample 2) .

## Results

### Preliminary Analyses

Descriptive statistics and bivariate correlations are presented in Table 1 (for Sample 1) and in Table 2 (for Sample 2). A multivariate analysis of variance (MANOVA) showed significant gender differences in Sample 1 (Wilk’s Λ = .919, *F*[5, 209] = 3.71, *p* < .01, multivariate η^2^ = .08). A follow-up analysis of variance (ANOVA) with Bonferroni correction showed that males scored lower than females in fear of failure *F*(1, 213) = 13.27, *p* < .001, η^2^ = .06 (*M_male_* = 2.81, *SD* = 0.97 vs. *M_female_* = 3.30, *SD* = 0.90). A MANOVA also showed significant gender differences in Sample 2 (Wilk’s Λ = .836, *F*[11, 166]= 2.97, *p* < .001, multivariate η^2^ = .16). A follow-up ANOVA with Bonferroni correction showed that males scored lower than females in fear of failure, *F*(1, 176) = 7.89, *p* < .05, η^2^ = .00 (*M_male_* = 2.84, *SD* = 0.87 vs. *M_female_* = 3.15, *SD* = 0.84), controlling reasons underlying MAp goals, *F*(1, 176) = 4.55, *p* < .05, η^2^ = .03 (*M_male_* = 3.02, *SD* = 0.95 vs. *M_female_* = 3.32, *SD* = 0.84), as well as MAv goals, *F*(1, 176) = 4.11, *p* < .05, η^2^ = .02 (*M_male_* = 3.76, *SD* = 0.73 vs. *M_female_* = 3.97, *SD* = 0.63). Therefore, gender was included as a covariate in the subsequent analyses in both samples.

**Table 1:** Bivariate Correlations of the Measured Variables (Sample 1).

	1	2	3	4	5	6	7	8	9	10	11	12

*Background variables*												
1. Age	–											
*Antecedents*												
2. Motive to suceed	.04	–										
3. Motive to avoid failure	–.04	.10	–									
4. Need satisfaction	.00	.22**	–.05	–								
5. Need frustration	–.15*	.05	.31**	.10	–							
*Motivational variables*												
6. MAp goals	–.09	.21**	–.01	.19**	–.01	–						
7. MAp autonomous	.11	.33**	.13	.38**	.10	.41**	–					
8. MAp controlling	–.06	.14*	.32**	.20**	.25**	.20**	.51**	–				
9. MAv goals	–.06	.08	.06	.21**	.16*	.32**	.25**	.17*	–			
10. MAv autonomous	.09	.16*	.18**	.19*	.16*	.19*	.60**	.42**	.57^**^	–		
11. MAv controlling	.02	.15*	.31**	.17*	.33**	.04	.42**	.65**	.30^**^	.62^**^	–	
*Educational outcomes*												
12. Learning strategies	.19**	.33**	.01	.23**	.01	.27**	.42**	.21**	.21**	.31**	.16*	–
*M*	22.36	4.30	3.20	3.60	2.90	4.30	3.90	3.17	3.54	3.60	2.80	3.44
*SD*	3.92	0.59	0.94	0.60	0.74	0.83	0.74	1.08	1.11	0.95	1.04	0.61

*Note.* * *p* < .05. ** *p* < .01. MAp = Mastery-approach goals; MAv = Mastery-avoidance goals.

**Table 2:** Bivariate Correlations of the Measured Variables (Sample 2).

	1	2	3	4	5	6	7	8	9	10	11	12	13

*Background variables*													
1. Age	–												
*Antecedents T1*													
2. Motive to suceed	.08	–											
3. Motive to avoid failure	.05	.04	–										
4. Need satisfaction	.06	.25**	–.10	–									
5. Need frustration	.14*	.07	.36**	.10	–								
*Motivational variables T1*													
6. MAp goals	.08	.21**	.04	.18**	–.12	–							
7. MAp autonomous	.08	.20**	.07	.16**	.02	.32**	–						
8. MAp controlling	.14*	.15*	.31**	.05	.32**	.10	.51**	–					
9. MAv goals	.08	.14*	.13*	.15*	.02	.22**	.25**	.25**	–				
10. MAv autonomous	.07	.24**	.15**	.15*	.13	.26**	.54**	.40**	.34**	–			
11. MAv controlling	.15*	.22**	.30**	.09	.38**	.07	.31**	.60**	.27**	.72**	–		
*Educational outcomes T1 & T2*													
12. Learning strategies T1	.13*	.23**	.14*	.20**	.03	.34**	.40**	.10	.22**	.28**	.12	–	
13. Learning strategies T2	.15*	.21**	.16*	.32**	.03	.43**	.41**	.17*	.24	.29**	.12	.67^**^	–
*M*	19.54	4.14	3.03	3.43	2.85	4.22	3.58	3.21	3.47	3.15	2.96	3.19	3.20
*SD*	1.50	0.65	0.87	0.53	0.69	0.74	0.79	0.93	1.10	0.81	0.88	0.54	0.53

*Note.* * *p* < .05. ** *p* < .01. MAp = Mastery-approach goals; MAv = Mastery-avoidance goals.

### Main Analyses

**Aim 1: Application of reasons underlying MAv goals.** Inspection of the cumulative percentages showed that 89.3% (Sample 1) and 84.2% (Sample 2) of the students scored equal or higher than 3 with respect to their autonomous reasons for pursuing MAp goals, whereas 61.2% and 66.8% of the students scored equal or higher than 3 with respect to controlling reasons underlying MAp goals. Regarding the autonomous reasons underlying MAv goals, 77.9% and 68.1% scored equal or higher than 3, whereas regarding the controlling reasons underlying MAv goals, 51.6% and 63.1% of the participants scored equal or higher than 3. It seems that in both MAp and MAv goals high controlling reasons were reported by less students in comparison to autonomous reasons. However in both cases, more than 50% of the participants had a high score in controlling reasons, indicating that autonomous and controlling reasons are applicable to MAp and MAv goals.

**Aim 2: Predictive validity of reasons underlying MAv goals.** We performed a hierarchical regression analysis to explore the relations of MAv goals and their underlying reasons with the learning strategies. Specifically, we regressed learning strategies onto the MAv goals in Step 1 and their underlying reasons in Step 2 while controlling for MAp goals in Step 3. In Step 1 (*F*[1, 142] = 12.13, p < .01, adjusted *R*^2^ = .07) MAv goals initially predicted the use of learning strategies (β = .28, *p* < .01), but in Step 2 (*F*[2, 140] = 4.24, p < .05, adjusted *R*^2^ = .11) this effect disappeared (β = .12, p > .05) when the autonomous (β = .32, *p* < .01) and controlling reasons (β = –.08, *p* > .05) for MAv goal pursuit were added. In Step 3 (*F*[1, 139] = 7.82, p < .01, adjusted *R*^2^ = .15), autonomous reasons remained significant (β = .26, *p* < .05) next to MAp goals (β = .23, *p* < .01), while Mav goals (β = .06, *p* > .05) and their controlling reasons (β = –.03, *p* > .05) were nonsignificant. No significant interactions between MAv goals and controlling or autonomous underlying reasons were found in the prediction of learning strategies. We performed a similar analysis for Sample 2, with T2 learning strategies as an outcome. Different from Sample 1, neither MAv goals, nor their underlying reasons, nor MAp goals predicted learning strategies six weeks later and the same was true for the interactions between MAv goals and both types of underlying reasons.

**Figure 2:**
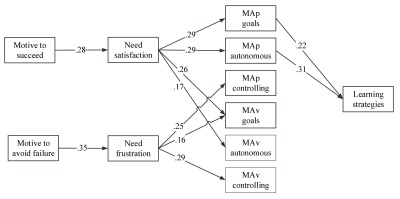
The Tested Model of Sample 1 (*Cross-sectional Study*) controlling for gender differences (not shown for sake of clarity). Also, not shown for sake of clarity are the correlations among MAp, MAv goals and autonomous or controlling underlying reasons.

**Aim 3: Testing an integrated model.** We performed path analysis using EQS 6.1 structural equation modeling statistical software package (Bentler, 1995) to investigate both the explanatory role of need-based experiences as well as the “what” and “why” of achievement goals in the relation between the achievement motives and learning strategies. All the constructs were represented by the mean score of the measured variable with the exception of learning strategies that was defined by the mean of metacognitive self-regulation, critical thinking, and effort regulation. We considered it to be important to use the mean of the three learning strategies to reduce the number of variables and to run a path analysis with the 158 students of Sample 2 who participated at both waves. To keep the two models parallel and comparable, we followed the same logic (i.e., a mean of the three learning strategies) for the model tested in Sample 1. As can be noticed in Figure 2, all hypothesized paths from Sample 1 model were significant and fit indices were acceptable: S-Bχ2 (31, *N* = 141) = 46.85, *p* < .05, CFI = .966, SRMR = .088, RMSEA = .060 (90%-CI: .017 – .094). A test of indirect effects showed the following: the motive to succeed was indirectly associated with MAp goals (*β* = .08, *z* = 2.11, *p* < .05), MAp autonomous reasons (*β* = .08, *z* = 2.63, *p* < .01) and MAv goals (*β* = .07, *z* = 2.12, *p* < .05) by means of need satisfaction, whereas the motive to avoid failure was indirectly associated with MAp controlling (*β* = .09, *z* = 2.648, *p* < .01) and MAv controlling reasons (*β* = .10, *z* = 3.23, *p* < .01) via need frustration. Also, indirect effects showed that the motive to succeed and need satisfaction were indirectly associated with learning strategies (*β* = .04, *z* = 2.38, *p* < .05 and *β* = .15, *z* = 3.91, *p* < .01 respectively) via MAp goals and MAp autonomous reasons. It is important to clarify that direct paths from the motive to avoid failure and the motive to succeed to mastery goals and their underlying reasons as well as to learning strategies were allowed, but none of these paths were significant and therefore were dropped from the model.

**Figure 3:**
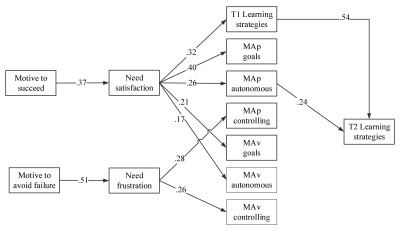
The Tested Model of Sample 2 (*Short-longitudinal Study*) controlling for gender differences (not shown for sake of clarity). Also, not shown for sake of clarity are the correlations among MAp, MAv goals and autonomous or controlling underlying reasons.

In Sample 2, as it is shown in Figure 3, the hypothesized paths were also significant with the exception of the path from MAp to learning strategies, which was then dropped from the model. The final model yielded acceptable fit indices: S-Bχ2 (34, *N* = 103) = 52.68, *p* < .05, CFI=.954, SRMR = .084, RMSEA = .073 (90%-CI: .029 – .110). A test of indirect effects showed that the motive to succeed was indirectly associated with MAp goals (*β* = .15, *z* = 3.35, *p* < .05), MAp autonomous reasons (*β* = .09, *z* = 2.46, *p* < .05), MAv goals (*β* = .08, *z* = 2.53, *p* < .05), MAv autonomous reasons (*β* = .06, *z* = 2.47, *p* < .05) and learning strategies in T1 (*β* = .12, *z* = 2.56, *p* < .05) by means of need satisfaction, whereas the motive to avoid failure was indirectly associated with MAp controlling (*β* = .14, *z* = 3.49, *p* < .01) and MAv controlling reasons (*β* = .13, *z* = 2.92, *p* < .01) via need frustration. Also, a test of indirect effects showed that the motive to succeed and need satisfaction were indirectly associated with learning strategies in T2 (*β* = .08, *z* = 2.57, *p* < .51 and *β* = .23, *z* = 3.23, *p* < .01 respectively) via MAp autonomous reasons and learning strategies in T1.

## Discussion

The present study tried to build upon the enriched hierarchical model of achievement motivation (Michou, Vansteenkiste et al., 2014) by having three main purposes. Its first purpose was to investigate whether autonomous and controlling reasons are meaningful in the adoption of MAv goals. The second purpose was to investigate the relations of MAv goals and their underlying reasons to learning strategies when MAp goals and their underlying reasons were also considered. Its third purpose was to incorporate need-based experiences as explanatory variables in between the motive to succeed and avoid failure and both the “what” and “why” of achievement goals.

### Autonomous and Controlling Reasons Underlying MAv Goals

The findings of the present study showed that MAv goals, much similar as MAp goals, can be endorsed for either autonomous or controlling reasons. But what is the meaning of this result in the prediction of students’ learning strategies? In Sample 1, when we isolated the MAv goals and their underlying reasons as predictors of learning strategies, the autonomous reasons were found to matter over and above the MAv goals as such. This finding indicates that the reasons underlying MAv goals can further explain the relation of students’ motivation to educational outcomes and therefore, when both the MAv goals and their underlying reasons are considered, a more refined insight of the achievement motivation can be attained.

However, the predictive power of these autonomous reasons changed as a function of the inclusion of additional predictors. That is, when controlling for MAp goals, the initial contribution of autonomous reasons of MAv goals was somewhat reduced and it was reduced even more when the reasons underlying MAp goals were also included in the path analysis. Specifically, only MAp goals and its underlying autonomous reasons related positive to learning strategies. Also in previous research both MAp goals and their autonomous underlying reasons were related to positive outcomes (Gaudreau, 2012). However, this previous research focused on the predictive value of either MAp or PAp goals and their underlying reasons, leaving the question unaddressed whether the observed effects of either the “what” or the “why” of achievement striving will remain when the endorsement of other achievement goals and their underlying reasons will be taken into consideration. In our study, we examined the relation of both mastery goals and their underlying reasons to learning strategies and observed that only MAp goals and MAp autonomous reasons related positively to effective learning.

Additionally, in Sample 2, only autonomous reasons for pursuing MAp goals predicted learning strategies in T2, which suggests that, eventually, the autonomous reasons for pursuing MAp goals could have a more lasting predictive value for learning strategies than the goal itself. This finding needs further investigation as, to our knowledge, only one more longitudinal study has examined the effects of an achievement goal (i.e., PAp goal) and its underlying reasons on affective outcomes few months later (Gillet et al., 2012).

Taking together the results of both samples, our study seems to show that students’ MAp goals and the autonomous reasons behind them are stronger positive predictors of adaptive learning strategies than the MAv goals and their underlying autonomous reasons. These results suggest that there is a differentiation in the predictive value of achievement goals and underlying reasons to educational outcomes according to their content and indicate the importance of considering both the achievement goals and their autonomous and controlling reasons in the prediction of learners’ outcomes. Moreover, in terms of the educational implications of our findings, it seems that while it is important for teachers to promote MAp goals, it is even more important to communicate in an autonomous supportive way with their students so that students can endorse an achievement goal for autonomous reasons.

### Need Satisfaction and Frustration as Explanatory Mechanisms

Previous studies have shown that the approach and avoidance valence of success and failure, as it is manifested in the achievement goals, is the product of a dispositional motive to succeed or to avoid failure formed in the individual’s early years (Elliot, 1999; Elliot & Church, 1997). Previous studies have also shown that the motive to succeed could be related to autonomous motivation, whereas the motive to avoid failure could be related to controlled motivation (Michou, Matsagouras et al., 2014). Additionally, motive dispositions have been related to achievement motivation considered as the goal complex of an achievement goal with its underlying reasons (Michou, Vaanstenkiste et al., 2014). We deemed timely in the present study to build upon the previous findings by investigating need satisfaction and need frustration as the mechanism through which the achievement motives relate to achievement goals and underlying reasons and, through them, to learning outcomes.

In this investigation we took need satisfaction and frustration as they have been widely considered: the prerequisite of the autonomous and controlled motivation respectively – the “why” aspect of achievement goals. For this reason, in our study need satisfaction and frustration in university studies (i.e., contextual level) are considered as the antecedent of the achievement goals and underlying reasons in a specific university (or preparatory) course (i.e., situational level). The results of our study uncovered a potential mediating role of need satisfaction and frustration between motive dispositions and goal complexes.

As it was expected, need satisfaction mediated the relation between the motive to succeed and autonomous reason underlying achievement goals. Alternatively, need frustration mediated the relation between controlling reasons for pursuing achievement goals and the motive to avoid failure. More interestingly, the motive to succeed was positively related to MAp and MAv goals via need satisfaction whereas the motive to avoid failure was positively related to MAv goals via need frustration (yet only in Sample 1). Need satisfaction and need frustration can be considered not only the predictors of autonomous and controlled motivation, but also as the antecedents of achievement goals. This finding extends our understanding of achievement motivation and shows that autonomous and controlling regulations are ideal to be considered as the “why” aspect of achievement goals given that they share common motivational antecedents.

It seems that the motive to succeed could predispose students to perceive need satisfaction, whereas the motive to avoid failure could predispose students to perceive need frustration in their educational environment. Educational environments are considered the most achievement oriented contexts. A student with a high desire to succeed (and a low fear of failure) could feel in harmony with the achievement settings in school, college or university. Consequently, she could undertake a more agentic role in the fulfillment of her psychological needs, increasing thus the likelihood to satisfy them (Reeve, 2013). In sequence, need satisfaction could be related to autonomous reasons for pursuing either an approach (e.g., MAp) or an avoidance (e.g., MAv) goal and to these goals as well. In contrast, a student with a high motive to avoid failure (and low motive to succeed) could feel threatened in an achievement orientated educational setting. For this reason, he or she may be more likely to undertake a more defensive role in the fulfillment of his or her psychological needs, therefore perceiving others as more powerful. This could be the basis of a perceived need frustration and in sequence, for controlling reasons underlying achievement goals as well as for the adoption of an avoidance goal.

The positive relations of the MAp goals and the autonomous reasons underlying MAp and MAv goals with adaptive learning outcomes reveal the importance of both motive to succeed and need satisfaction in the prediction of positive educational outcomes. To state it differently, it seems that both individual differences and need satisfaction and frustration have to be considered in understanding students’ achievement motivation and outcomes. In research undertaken in the framework of SDT, individual differences are not always considered, as need satisfaction has been proven beneficial for all humans’ optimal functioning irrespective of personality traits. However, personal dispositions seem to color people’s perceptions of need satisfaction or frustration (see also Vallerand, 2000). Thus, taking into consideration individual differences, we could study in a refined fashion who is more likely to satisfy his or her needs. It is obvious that further research is needed to test this assumption and we believe that the enriched hierarchical model of achievement motivation, suggested by Willy Lens, Maarten Vansteenkiste and collaborators, provides a concrete framework for such research.

### Future Directions

At this point, we considered it to be important to summarize our suggestions for future directions in research related to the “what” and the “why” of achievement striving. Specifically, we believe that longitudinal studies can provide further evidence about the long-term predictive value of both the MAp goals and their underlying autonomous reasons. In addition, further evidence about the predictive value of MAp goals and their underlying reasons could be provided if the task-related (i.e., to complete correctly a task) and self-related (i.e., to improve one’s own performance) distinction of MAp goals suggested by Elliot, Murayama, and Pekrun (2011) were going to be tested under the lens of “the what” and “the why” of achievement striving (but see Delrue et al., this issue). Finally, an investigation about the possibility that the motive to succeed and the motive to avoid failure may color people’s perceptions about need satisfaction and their benefits from need-supportive environments could further extend our understanding of achievement motivation.

### Limitations

The cross-sectional and short prospective designs of the studies prevent us from claiming cause-effect phenomena. Experimental studies or long-term longitudinal studies are needed to test the causal relationships among the dispositional achievement motives, need satisfaction and frustration, the achievement goals and their underlying reasons, and educational outcomes. Regarding the content of our study, there are three limitations: (a) the endorsement of MAp or MAv goals was assessed by only one item and internal consistency cannot be reported, (b) the controlling reasons were assessed only by introjected reason-items as the external reason-item failed to load on the controlling factor, and (c) the internal consistency for learning strategies was in some cases rather low. Also further research is needed in order to generalize the results to other cultures and contexts. Up to now, the enriched hierarchical model of achievement motivation has been tested in Greek and Turkish samples of students. Researches in more individualistic cultures and in other than educational achievement settings (e.g., sport or work) are also needed.

## Conclusion

The enriched hierarchical model of achievement motivation proposed by Willy Lens and his associates provides a framework to extend our knowledge on achievement motivation. In our study, based on this model, we found that the endorsement of MAv goals can be regulated by both autonomous and controlling reasons. Furthermore, in our study, we found that MAp goals and their underlying reasons predicted learning strategies when MAv goals and their underlying reasons were also considered. This finding suggests that the consideration of only one achievement goal and its underlying reasons in the prediction of outcomes can be misleading regarding whether the observed effects of either the “what” or the “why” still exist when other achievement goals are also endorsed for the same or different reasons. Our last conclusion is that need satisfaction and frustration at the contextual level mediated the relation between personal dispositional antecedents and achievement goals and their underlying reasons. It seems that both personal and contextual antecedents of achievement motivation are useful aspects to be considered especially when interventions in the achievement settings are designed to improve achievers’ quality of motivation and outcomes.

## Footnotes

Performance goals were also divided into *Performance-approach goals* (PAp) that orient individuals to reach a normative-based success by outperforming others (e.g. my goal is to get a better grade compared to my classmates), and *performance-avoidance goals* (PAv) that orient individuals to avoid a normative-based failure (e.g. my goal is to avoid performing worse than my classmates) (Elliot & McGregor, 2001).
